# The Healing Effect of Grape Seed Oil Enema with or without Sesame Oil in Acetic Acid Induced Ulcerative Colitis of Rats

**Published:** 2017-05

**Authors:** Fatemeh Hosseinzadeh, Moosa Salehi, Nader Tanideh, Davood Mehrabani, Azadeh Sayarifard, Anahita Sedighi

**Affiliations:** 1 Department of Clinical Nutrition, School of Nutrition and Food Sciences, Shiraz University of Medical Sciences, Shiraz, Iran;; 2 Nutrition and Food Sciences Research Center, Shiraz University of Medical Sciences, Shiraz, Iran;; 3 Stem Cell Technology Research Center, Shiraz University of Medical Sciences, Shiraz, Iran;; 4 Department of Pharmacology, School of Medicine, Shiraz University of Medical Sciences, Shiraz, Iran;; 5 Center for Academic and Health Policy, Tehran University of Medical Sciences, Tehran, Iran;; 6 Department of Cellular and Molecular Biology, Pharmaceutical Science Branch, Islamic Azad University, Tehran, Iran

**Keywords:** Grape seed oil, Sesame oil, Ulcerative colitis, Enema

## Abstract

**BACKGROUND:**

Inflammatory bowel diseases contain two digestive system diseases, ulcerative colitis (UC) and Crohn’s disease with unclear causes. The aim of present study was to investigate the therapeutic effects of administration of the Sesame oil (SO) and grape seed oil (GSO) as enema route in rats suffering from experimental acetic acid induced UC.

**METHODS:**

Eighty male rats were randomly allocated into 8 equal groups as health control (HC_1_) without any disease treated with 1 ml of normal saline as enema; HC_2_ received SO; HC_3_ received GSO; negative control (NC) with induced UC receiving 1 ml of normal saline as enema; and positive control (PC) with induced UC treated by asacol. All treatments were performed identically with 4 mg/kg of medication except for asacol that was 100 mg/kg for 7 days. The weight changes was recorded after seven days. The serum levels of malondialdehyde (MDA), total antioxidant capacity (TAC), interleukin-6, and c-reactive protein (CRP) were measured. Colon macroscopic and microscopic histological changes were also measured at the end of 7^th^ day.

**RESULTS:**

No significant changes were detected in weight in neither groups on day 0 nor at the end of study. No beneficial effects were seen for all treatments regarding healing process and the decrease in inflammation. Between treatment groups, the lowest MDA (7.40±0.98 U/ml), CRP (83.20±10.01 mg/l) and IL-6 levels (130.86±10.70 mU/ml) and highest TAC (1.91±0.43 mmol/l) belonged to GSO group.

**CONCLUSION:**

GSO enema alone can be considered as a treatment of choice for UC due to its antioxidant properties.

## INTRODUCTION

Inflammatory bowel diseases (IBDs) contain two digestive system diseases, ulcerative colitis (UC) and Crohn’s disease (CD) with unclear causes. The occurrence of UC can be affected by environmental, genetics, immune factors, and gut microbiota.^1^ UC is demonstrated by mucosal inflammation and limited to the colon. A significantly reduced biodiversity in fecal microbial count and diversity in IBD patients compared to those in healthy controls were reported.^1^^,^^2^ It has been reported that high intakes of mono- and polyunsaturated fats and vitamin B6 are associated with an increased risk to develop UC.^3^

So it can be suggested that use of any chemical or herbal agents in enema route can be effective in treatment of UC by changing colon microbiome and also by producing local effects. There are several ways to induce UC in experimental animals such as use of acetic acid as acetic acid is inexpensive and available in most laboratories.^4^ Grape is a fruiting berry of the deciduous woody vines of the botanical genus *Vitis*.^5^ Grape seed oil (GSO) has many uses ranging from cooking (as a food additive), cosmetics and in controlling several diseases and wound healing potential.^6^ Grape seed and GSO are rich in unsaturated fatty acids and antioxidants such as tocopherols, phytosterol, and α- and γ-tocotrienol.^7^

Although oil content and fatty acid composition of grape seed show great variation among various genotypes,^8^ but it has been reported that the predominant fatty acids in the GSO were linoleic (65,0%), linolenic (1,5%), oleic (17,0%), and palmitic (8,0%) acids which paly significant roles in lipid metabolism.^9^ Unlike GSO, sesame oil (SO) is a known oil which showed any therapeutic effects due to its antioxidant, antibacterial and anti-inflammatory agents.^10^^,^^11^ There are no reports about the use of GSO with or without SO in the acetic acid induced UC neither in enema route application nor by other routes. Therefore, the aim of the present study was to evaluate the healing effect of GSO alone or in combination with SO in enema route in the acetic acid induced ulcerative colitis. 

## MATERIALS AND METHODS

The use of experimental animals in this study was approved by Ethical Committee of Shiraz University of Medical Sciences. All efforts were made to minimize animal stress and handling and also the animal welfare was under consideration in all part of the study. Eighty male Sprague Dawley rats (200±20 g) were obtained from the Center of Experimental and Comparative Medicine, Shiraz University of Medical Sciences, Shiraz, Iran. The rats were randomly allocated into 8 equal separated groups as follow and were housed in standard cages under a 12-h light cycle (lights on at 7:00 pm) with an ambient temperature of 22±2°C, and 55% relative humidity.

Healthy control I (HC_1_) group did not undergo induction of UC and received 1 ml of normal saline by enema route, HC_2_ group did not undergo UC induction and received 4 mg/kg of SO, HC_3 _group did not undergo UC induction and received 4 mg/kg GSO, Negative control (NC) group underwent UC induction and received 1 ml of normal saline by enema route, Positive control (PC) group underwent UC induction and received 100 mg/kg of asacol by enema route, SO group underwent UC induction and received 4 mg/kg of SO by enema route, GSO group underwent UC induction and received 4 mg/kg of GSO by enema route, and SO+GSO group that underwent UC induction and received both 4 mg/kg SO and 4 mg/kg GSO by enema route.

Oils used in this study were Zareentalia Grap seeds Oil (Zareentalia Co, Italy) and Samar sesame oil (Samar Co, Iran). These oils were produced from the best quality raw materials and having the standard production certificates. All animals were fasted overnight and their bowels were cleaned before induction of UC to emptying the colon. UC induction was performed using 2 ml of 3% acetic acid transrectally as described before.^4^ All treatment were applied in enema route. 

The weight changes were recorded before the study (day 0) and at the 7^th^ day (end of the study) by using a digital scale with 0.1 g precision. At the end of the seventh days, the rats were sacrificed in the CO_2_ induction box. Then, the distal 8 cm of the colon was removed and dissected by longitudinal incision. We processed, stained and evaluated the colon tissues of rats in each groups as reported before.^12^ Indices which presented by Morris et al.^13^ as 0-5 degree for macroscopic mucosal injuries, by Murthy et al.^14^ as 0-4 degree for crypt injuries, and Onderdonk et al.^15^ as 0-3 degrees for level of inflammation were used in this study to evaluate the histopathological changes of the colon tissues. 

The serum samples were separated from the heart blood using centrifugation (3000 rpm, 10 min). The malondialdehyde (MDA) level, as the end-products of lipid peroxidation (LPO) and one of the most important indices of oxidative stress, was assessed via the measurement of thiobarbituric acid reactive substances (TBARS) in sera.^16^ In addition, the total antioxidant capacity (TAC) in serum was evaluated by colorimetric method using commercial kit (Cayman, USA). The IL-6 content of the sera was measured using rat specific sandwich ELISA kit (Sigma Aldrich, USA). Serum CRP was evaluated by enzyme immunoassay kit (IBL international, Germany).

All of our evaluated variables were biological parameters and have normal distribution in the population. Therefore, we can face with it as parametrical variables. The data are presented as mean and standard deviation. Normality of data were checked by Kolmogorov-Smirnov test. One-way ANOVA and Tukey post hoc test were used for comparison of mean differences in all variables between 8 groups. *p*<0.05 was considered statistically significant. Mann-Whitney U test with Bonferroni correction was used for comparing histopathology scores between different groups on different days. SPSS 16.5 software was used to analyze the data.

## RESULTS

There are no significant differences in weight of rats between different groups in neither day 0 nor 7^th^ day (*p*>0.05). However, the highest mean weight belonged to GSO group (187.63±9.74 g). The mean and SD of macroscopic and microscopic scores in different groups are presented in Table 1. As shown, NC and then SO+GSO had highest degree of macroscopic lesions which significantly differed from HC_1_ group (*p*<0.001 and *p*=0.005, respectively). The highest microscopic lesions and inflammation severity were seen in NC group which significantly higher than all other 7 groups (*p*<0.05). No significant differences were detected in these variables between other 7 groups (*p*>0.05).

**Table 1:** Wight changes, macroscopic and microscopic evaluations of the colon tissue in different groups

**Groups**	**Weight (day 0)**	**Weight (day 7)**	**Morris** ^13^	**Onderdonk** ^15^	**Murthy** ^14^
HC_1_	168.60±9.87^a^	178.20±10.47^a^	0.00±0.00^b^	0.00±0.00^a^	0.00±0.00^a^
HC_2_	176.00±3.16^a^	176.00±7.07^a^	0.60±0.89^ab^	0.60±0.55^a^	0.60±0.55^a^
HC_3_	174.40±9.40^a^	171.80±11.34^a^	1.60±1.82^bc^	0.20±0.45^a^	0.20±0.45^a^
NC	177.22±9.90^a^	167.22±19.29^a^	3.00±1.32^a^	2.67±0.87^b^	2.44±0.53^b^
PC	165.67±8.04^a^	166.67±10.39^a^	0.33±0.52^bc^	0.00±0.00^a^	0.00±0.00^a^
SO	172.38±11.20^a^	172.00±20.20^a^	1.75±1.58^ab^	0.63±1.19^a^	0.75±1.39^a^
GSO	176.63±11.61^a^	187.63±9.74^a^	0.88±0.83^bc^	0.88±0.35^a^	0.88±0.35^a^
SO+GSO	169.29±4.61^a^	172.86±20.19^a^	2.43±1.90^ca^	1.00±1.15^a^	1.29±1.50^a^

HC1 group: Neither UC nor treatment; HC2 group: No UC induction but use of 4 mg/kg sesame oil (SO); HC3 group: No UC induction but use of 4 mg/kg grapeseed oil (GSO); NC group: UC induction without treatment; PC group: UC induction but treated with 100 mg/kg Asacol; SO group: UC induction but treated with 4 mg/kg SO; GSO group: UC induction but treated with 4 mg/kg GSO; SO+GSO group: UC induction but treated with both 4 mg/kg SO and 4 mg/kg GSO. Different superscript letters in each column indicate significant difference (*P*<0.05).

**Fig. 1:**
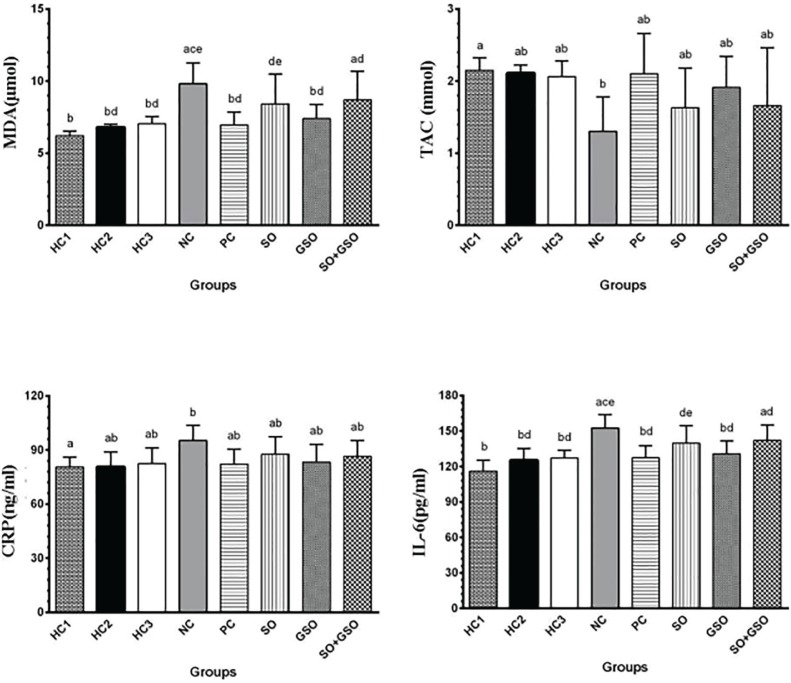
Mean and SD of oxidative stress indices, inflammation markers in different groups after seven days treatment. HC1 group: Neither UC nor treatment; HC2 group: No UC induction but use of 4 mg/kg sesame oil (SO); HC3 group: No UC induction but use of 4 mg/kg grapeseed oil (GSO); NC group: UC induction without treatment; PC group: UC induction but treated with 100 mg/kg Asacol; SO group: UC induction but treated with 4 mg/kg SO; GSO group: UC induction but treated with 4 mg/kg GSO; SO+GSO group: UC induction but treated with both 4 mg/kg SO and 4 mg/kg GSO. Significant differences between groups in each variable are demonstrated by different superscript letters (P<0.05).

The mean and SD of oxidant and antioxidant status, inflammatory indices in the serum at the end of the study period (day 7) are presented in Figure 1. Between treatment groups, the lowest MDA (7.40±0.98 U/ml), CRP (83.20±10.01 mg/l) and IL-6 levels (130.86±10.70 mU/ml) and highest TAC (1.91±0.43 mmol/l) were belonged to GSO group. This demonstrated the higher efficacy of GSO in treatment of UC and prevention of oxidative stress and inflammation.

**Fig. 2:**
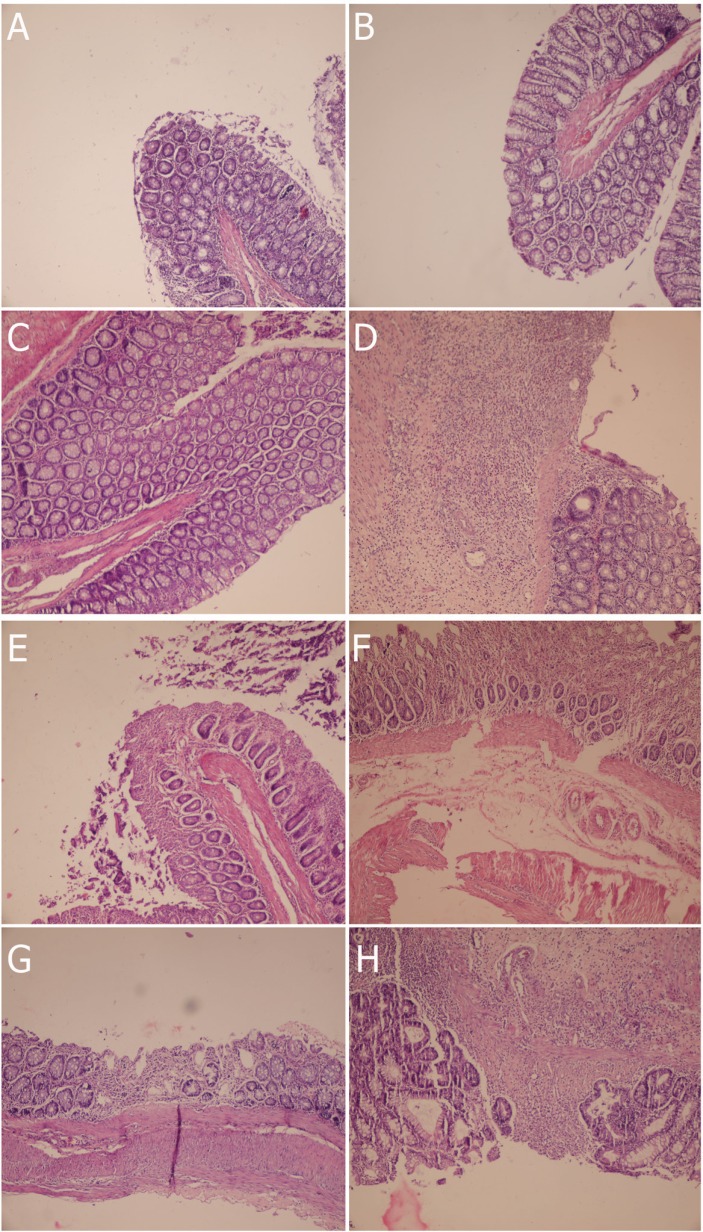
Histopathological changes in colon tissue in different groups. A, HC1 group (No UC with 1ml enema normal saline treatment); B, HC2 group (No UC induction but use of 4 mg/kg SO); C, HC3 group (No UC induction but use of 4 mg/kg GSO); D, NC group (UC induction with 1ml enema normal saline treatment); E, PC group (UC induction but treated with 100 mg/kg Asacol); F, SO group (UC induction but treated with 4 mg/kg SO); G, GSO group (UC induction but treated with 4 mg/kg GSO); H, SO+GSO group (UC induction but treated with both 4 mg/kg SO and 4 mg/kg GSO

The histopathological features of colon tissue are presented in Figure 2. As shown, control groups showed no histopathological changes. But negative control showed typical ulcer which near completely healed in response to asacol (positive control) and partially in response to SO, GSO and their combination.

## DISCUSSION

UC as one type of IBD has several treatment methods which are anti-inflammatory and immune modulating drugs such as salazosulfapyridine, mesalazine, corticosteroids, azathioprine, 6-mercaptopurine, methotrexate and cyclosporine.^17^ However, applying new treatments needs prior animal studies and finding good model of UC induction. Also, several studies reported different animals as experimental models of UC.^18^^,^^19^

In the present study, we evaluated and compared the effects of GSO alone or in combination with SO in the healing of colon tissue suffered from acetic acid induced UC in rat. This performed by macroscopic and microscopic changes comparison, antioxidant status, and inflammatory indices evaluations. No beneficial effects were seen for all treatments in point of weight gain, macroscopic and microscopic healing and decreasing of inflammation severity. But use of GSO alone was decreased oxidative stress and combated with inflammation. Therefore, enema application of GSO can be presented as a drug of choice for treatment of UC. 

The major symptoms in the human and animal UC include diarrhea, rectal bleeding and weight loss. It has been reported that use of plants which have antibacterial and antioxidant activities could be prevent the weight loss and also enhanced the rat weight in animal model of UC. It seem that the weight changes in response to any agents may be due to treatment of the health of the colon ulcers and also improving the overall health conditions.^20^^-^^23^ However, we could not find significant changes between different groups in neither day 0 nor 7^th^ day. This may be due to the route of administration and also unknown molecular and hormonal pathways which need further clarification. 

No macroscopic and microscopic beneficial effects at the same time, having antioxidant and anti-inflammatory properties were detected in this study just for GSO. Although, in the previous studies, it had been cleared that SO contain antioxidant factors which combat with inflammation and free radicals,^24^^-^^26^ but we could not find similar properties. There are some reports which expressed that GSO had antioxidant effects but they are not in UC. For instance, it has been reported that pretreatment with GSO exhibited a protective role against diazinon toxicity in rat by the antioxidant role of its constituents.^27^

Its beneficial antioxidant properties against ethanol,^28^ tetrachloride carbon,^29^ and cyclophosphamide^30^ toxicities and also acrylamide induced lesion^31^ were also reported. All of these beneficial effects plus our findings in UC are related to natural antioxidants which existed in GSO as reported previously.^32^ We conclusively found beneficial effects from the use of GSO alone but not in combination with SO as enema route in induced UC in rat. This mostly related to antioxidative and anti-inflammatory effects and therefore, use of GSO in enema route can be advised in them. However, performing randomized controlled trial to confirm such effects is highly necessary.
